# Evidence of *Borrelia theileri* in Wild and Domestic Animals in the Kafue Ecosystem of Zambia

**DOI:** 10.3390/microorganisms9112405

**Published:** 2021-11-22

**Authors:** Yongjin Qiu, David Squarre, Yukiko Nakamura, Alice C. C. Lau, Lavel Chinyama Moonga, Naoko Kawai, Aiko Ohnuma, Kyoko Hayashida, Ryo Nakao, Junya Yamagishi, Hirofumi Sawa, Boniface Namangala, Hiroki Kawabata

**Affiliations:** 1 Division of International Research Promotion, International Institute for Zoonosis Control, Hokkaido University, N 20 W 10, Kita-ku, Sapporo 001-0020, Japan; h-sawa@czc.hokudai.ac.jp; 2 Wildlife Diseases Unit, Department of Veterinary Services, Ministry of Fisheries and Livestock, Lusaka P.O. Box 50060, Zambia; davidsquarre@yahoo.co.uk; 3 Division of Collaboration and Education, International Institute for Zoonosis Control, Hokkaido University, N 20 W 10, Kita-ku, Sapporo 001-0020, Japan; yukiko.n52@gmail.com (Y.N.); lavelmwanga@gmail.com (L.C.M.); kawai@czc.hokudai.ac.jp (N.K.); kyouko-h@czc.hokudai.ac.jp (K.H.); junya@czc.hokudai.ac.jp (J.Y.); 4 Laboratory of Wildlife Biology and Medicine, Department of Environmental Veterinary Sciences, Faculty of Veterinary Medicine, Hokkaido University, N 18 W 9, Kita-ku, Sapporo 060-0818, Japan; alicelau.cc@vetmed.hokudai.ac.jp; 5 Technical Office, International Institute for Zoonosis Control, Hokkaido University, N 20 W 10, Kita-ku, Sapporo 001-0020, Japan; aikoh@czc.hokudai.ac.jp; 6 International Collaboration Unit, International Institute for Zoonosis Control, Hokkaido University, Sapporo 001-0020, Japan; 7 Laboratory of Parasitology, Department of Disease Control, Faculty of Veterinary Medicine, Hokkaido University, N 18 W 9, Kita-ku, Sapporo 060-0818, Japan; ryo.nakao@vetmed.hokudai.ac.jp; 8 Division of Molecular Pathobiology, International Institute for Zoonosis Control, Hokkaido University, N 20 W 10, Kita-ku, Sapporo 001-0020, Japan; 9 One Health Research Center, Hokkaido University, N 20 W 10, Kita-ku, Sapporo 001-0020, Japan; 10 Department of Paraclinical Studies, School of Veterinary Medicine, The University of Zambia, Lusaka 10101, Zambia; b.namangala@unza.zm; 11 Laboratory of Systemic Infection, Department of Bacteriology I, National Institute of Infectious Diseases, Toyama 1-23-1, Shinjuku, Tokyo 162-8640, Japan; kbata@nih.go.jp

**Keywords:** *Borrelia theileri*, cattle, impala, Kafue national park, Zambia

## Abstract

Members of the genus *Borrelia* are arthropod-borne spirochetes that are human and animal pathogens. Vertebrate hosts, including wild animals, are pivotal to the circulation and maintenance of *Borrelia* spirochetes. However, information on *Borrelia* spirochetes in vertebrate hosts in Zambia is limited. Thus, we aimed to investigate the presence of *Borrelia* spirochetes in wild animals and cattle in Zambia. A total of 140 wild animals of four species and 488 cattle DNA samples from /near the Kafue National Park were collected for real-time PCR screening, followed by characterization using three different genes with positive samples. Five impalas and 20 cattle tested positive using real-time PCR, and sequence analysis revealed that the detected *Borrelia* were identified to be *Borrelia theileri,* a causative agent of bovine borreliosis. This is the first evidence of *Borrelia theileri* in African wildlife and cattle in Zambia. Our results suggest that clinical differentiation between bovine borreliosis and other bovine diseases endemic in Zambia is required for better treatment and control measures. As this study only included wild and domestic animals in the Kafue ecosystem, further investigations in other areas and with more wildlife and livestock species are needed to clarify a comprehensive epidemiological status of *Borrelia theileri* in Zambia.

## 1. Introduction

Members of the genus *Borrelia* are arthropod-borne spirochetes that target vertebrate hosts and use them as reservoirs to complete their life cycle. The genus comprises three groups: Lyme disease borreliae, relapsing fever borreliae, and reptile-associated borreliae [1,2]. Among these groups, Lyme disease borreliae and reptile-associated borreliae are transmitted by Ixodid (hard-bodied) ticks [1,2]. Relapsing fever borreliae are divided into four subgroups: Soft tick-borne relapsing fever (STBRF), Hard tick-borne relapsing fever (HTBRF), Louse-borne relapsing fever (LBRF), and Avian worldwide relapsing fever [3], in which most of the identified relapsing fever borreliae belong to STBRF and are transmitted by Argasid (soft-bodied) ticks [4]. In contrast, HTBRF is transmitted by Ixodid ticks, such as *Amblyomma*, *Haemaphysalis*, *Ixodes*, and *Rhipicephalus* [5,6,7,8]. Only *Borrelia recurrentis*, belonging to LBRF, is transmitted by the human body louse [9]. In addition, *Borrelia anserina* of the Avian worldwide relapsing fever is transmitted by *Argas* spp. [10]. Recently, a different classification was given in Margos et al. (2020), who divides the genus into three groups: *Ixodes*-transmitted borreliae (*Borrelia burgdorferi* sensu lato complex), Matestriate-tansmitted borreliae, and relapsing fever-associated borreliae [11]. The classification of the genus *Borrelia* is still controversial.

*Borrelia lonestari*, *Borrelia miyamotoi*, and *Borrelia theileri* are members of HTBRF, or of Matestriate-tansmitted borreliae in Margos’s classification. *Borrelia lonestari* was first identified in 1996 [7] and was initially considered pathogenic to humans in the southern United States [12,13]. However, subsequent research did not support the pathogenicity of *B. lonestari* in humans [14]. *Amblyomma* ticks and white-tailed deer are vectors and suspected reservoirs, respectively, for *B. lonestari* in North America [15,16,17]. *Borrelia miyamotoi* was confirmed in 2011 as a human pathogen [18]. *Ixodes* ticks are reported vectors of *Borrelia miyamotoi* in Asia, Europe, North America, and Russia [19], and deer may act as natural reservoirs for it [20]. *Borrelia theileri* is a causative agent of bovine borreliosis, identified in South Africa more than 100 years ago by Arnold Theiler, who first found the agent transmitted by *Rhipicephalus* sp. to cattle [21,22]. To date, *Borrelia theileri* has been reported in cattle, goats, sheep, and horses in Africa, North and South America, and Australia [23,24,25]. *Rhipicephalus* ticks, such as *R. microplus*, *R. annulatus*, *R. evertsi*, and *R. decoloratus,* are well-known vectors of *Borrelia theileri*. In addition, *Borrelia theileri* has recently been detected in head lice (*Pediculus humanus*) in the Republic of Congo [26].

Vertebrate hosts, including wild animals, act as reservoirs for *Borrelia* spirochetes and are crucial in the circulation and maintenance of them. For instance, Kumagai et al. (2018) discovered *Borrelia* spp. of the hard tick-borne relapsing fever borreliae in 25.9% of wild deer tested in Japan and suggested that wild deer could act as reservoir hosts for *Borellia* spp. [27]. Similarly, other studies have also reported the detection of *Borrelia lonestari* and antibodies against *Borrelia burgdorferi* in wild deer populations in America [16,28]. Furthermore, several serological studies have revealed that many species of African antelopes kept in zoos have antibodies against *Borrelia burgdorferi* [29,30,31]. However, only a few studies have been conducted to investigate *Borrelia* spirochetes in wild animals in Africa. For example, *Borrelia* infection was demonstrated in 9.2% of small mammals tested in West Africa [32], and *Candidatus* Borrelia fainii was detected in 27% of bats examined in Zambia [33]. In addition, to date, there has been no investigation of *Borrelia* in large wild animals in Africa.

We aimed to investigate the presence of *Borrelia* spirochetes in large and medium-sized wild animals and cattle in Zambia through molecular and phylogenetic analyses.

## 2. Materials and Methods

DNA samples extracted from the whole blood of wild animals and domestic cattle from two previous studies [34,35] were used to detect *Borrelia* in this study. A total of 140 DNA samples from wild animals, including 97 impalas (*Aepyceros melampus*), 37 hartebeests (*Alcelaphus buselaphus*), four lions (*Panthera leo*), and two wild dogs (*Lycaon pictus*), were previously collected in the greater Kafue ecosystem for investigating piroplasm diversity [34]. In addition, we used 488 cattle DNA samples with packed cell volume (PCV) value data from a previous African trypanosome investigation [35]. The cattle breed was mainly a cross between local breeds (Tonga and Baila) and exotic breeds (mostly Boran and Brahman). Thus, a total of 628 DNA samples from wild and domestic animals were screened for *Borrelia* spp.

Specific semiquantitative real-time PCR was used for the initial screening of *Borrelia* infection, using the THUNDERBIRD^®^ Probe qPCR Mix (TOYOBO, Osaka, Japan) and primers (Bor16S3F, 5′-AGCCTTTAAAGCTTCGCTTGTAG-3′; Bor16S3R, 5′-GCCTCCCGTAGGAGTCTGG-3′; Bor16S3P, 5′-6FAM-CCGGCCTGAGAGGGTGAACGG-TAMRA-3′), which were designed to amplify a 148-bp fragment of the 16S ribosomal RNA gene (16S rDNA) of *Borrelia*. The specificity of the real-time PCR system for detection of *Borrelia* spp. was previously tested on DNA samples from 347 bacterial species [36]. All real-time PCRs were performed using a LightCycler 96 (Roche Diagnostics GmbH, Mannheim, Germany). The DNA of *Candidatus* Borrelia fainii strain Qtaro isolated from the patient in our previous study [33] and UltraPureTM distilled water (Invitrogen, Waltham, MA) were used as positive and negative controls, respectively, for each test. Samples with a cycle threshold level of log-based fluorescence <36 (~10–20 copies of spacer) were labelled positive, as described previously [36].

All positive samples from the real-time PCR were used for subsequent characterization based on three genes: flagellin (*flaB*), hypoxanthine-guanine phosphoribosyltransferase (*hpt*), and 16S rDNA using conventional PCR with the primers listed in Table 1. Briefly, PCRs were conducted using Ex-Taq HS (Takara, Shiga, Japan) with the following conditions: 1 min denaturation step at 98 °C followed by 35 cycles of 94 °C for 30 s, an appropriate annealing temperature (Table 1) for 30 s, and 72 °C for 30 s (1 min 30 s for 16S rDNA), and a final extension step at 72 °C for 5 min. For the negative and positive controls, UltraPureTM distilled water and DNA from Ca. Borrelia fainii strain Qtaro were added, respectively, instead of template DNA. The resulting PCR products were electrophoresed on a 1.2% agarose gel stained with Gel-Red (Biotium, Hayward, CA, USA) and visualized with a UV trans-illuminator.

Sanger sequencing was performed using BigDye Terminator version 3.1 chemistry (Applied Biosystems, Foster City, CA, USA). Sequencing products were run on an ABI Prism 3500 Genetic Analyzer, according to the manufacturer’s instructions. The sequence data were assembled using ATGC software version 6.0.4 (GENETYX, Tokyo, Japan). The DDBJ/EMBL/GenBank accession numbers for the sequences obtained were as follows: *flab*, LC656216-LC656235; 16S rDNA, LC656236-LC656247; and *hpt*, LC656248-LC656262 (Supplemental Table S1). The phylogenetic relationships for each gene were analyzed using the neighbor-joining and maximum likelihood methods with 1000 bootstraps implemented in MEGA X [40].

Statistical analysis was performed using GraphPad Prism 8 (GraphPad Software Inc., San Diego, CA, USA). For comparison of *Borrelia* spp. infection state in cattle and PCV value data of the samples, statistical significance (*p* < 0.05) was assessed using the Mann–Whitney test.

## 3. Results

### 3.1. Real-Time and Conventional PCRs

Five out of 97 (5.1%) impala samples tested positive for the real-time PCR, while none of the samples from other wild animal species tested positive (Table 2). Subsequent PCRs for the characterization of detected *Borrelia* spp. were conducted using five positive impalas, and PCRs targeting *flaB*, 16S rDNA, and *hpt* genes successfully provided amplicons from four, two, and four impalas, respectively. On the other hand, 20 out of 488 (4.1%) cattle samples tested positive for the real-time PCR (Table 2). Additional PCRs targeting *flaB*, 16S rDNA, and *hpt* genes successfully produced amplicons from 16, 10, and 11 cattle, respectively.

### 3.2. Sequence Analysis

The *flaB* sequences from the four impalas had two variants with one nucleotide difference in 276-bp. Variant 1 (Sample IDs: W2 and W97) and variant 2 (Sample IDs: W3 and W27) showed 100% (276/276 bp) and 99.6% (275/276 bp) identity, respectively, with *Borrelia theileri* strain KAT (KF569936). The 16S rDNA sequences were obtained from two impalas (Sample IDs: W2 and W97) and were identical. The sequence showed 99.6% (1350/1355 bp) identity with *Borrelia* sp. (AB897891) from *Haemaphysalis japonica*. The *hpt* sequences obtained from four impalas were identical and showed 99.7% (354/355 bp) identity with *Borrelia theileri* strain KAT (KF569937).

The *flaB* sequences from the 15 cattle samples (Sample IDs: B5, B8, B13, B33, B36, B38, B39, B44, B106, I16, I82, K23, K83, NN8, and Nt26) were identical and had the same sequence as that of variant 1 from two impalas (Sample IDs: W2 and W97). However, one cow (Sample ID: NN34) had one nucleotide difference in 276-bp and showed 99.6% (275/276 bp) identity with *Borrelia theileri* strain KAT (KF569936). The 16S rDNA sequences obtained from 10 cattle had tree variants. Variant 1 from eight cattle (Sample IDs: B33, B36, B38, B39, I16, I82, NN8, and Nt26) had the same sequence as impalas (Sample IDs: W2 and W97). Variant 2 (sample ID: B5) and variant 3 (sample ID: NN34) had one and two nucleotide differences in 1355-bp, respectively, from variant 1, and showed 99.6% (1349/1355 bp) and 99.5% (1348/1355 bp) identity with *Borrelia* sp. (AB897891) from *H. japonica*. The *hpt* sequences obtained from 11 cattle had two variants with one nucleotide difference in 389-bp. Variant 1 from 10 cattle (sample IDs: B5, B8, B33, B36, B38, B39, I16, I82, NN8, and Nt26) was identical to the sequence from the impalas. Variant 2 (Sample ID: NN34) showed 99.7% (352/353 bp) identity with *Borrelia theileri* strain KAT (KF569937).

### 3.3. Phylogenetic Analysis

**Figure 1:**
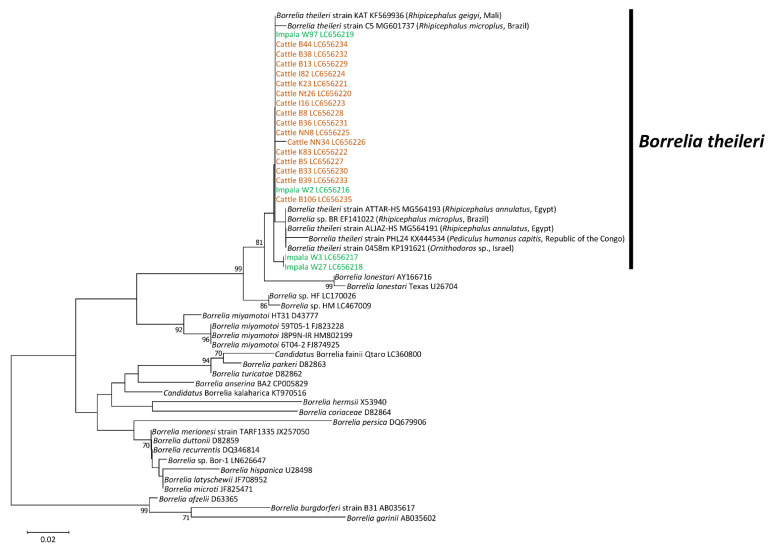
Phylogenetic inference of *Borrelia* spp. based on partial sequence of *flaB*. The accession numbers for nucleotide sequences are shown after the species names. The analysis was performed using the neighbor-joining method. Bootstrap values > 70% based on 1000 replications are presented on the interior branch nodes.

Phylogenetic trees were constructed to obtain information on the genetic association of our detected *Borrelia* spp. with other *Borrelia* species in the database. Based on the phylogenetic inference of the *flaB* gene, our detected *Borrelia* spp. from impalas and cattle were located within the clade of *Borrelia theileri* (Figure 1).

**Figure 2:**
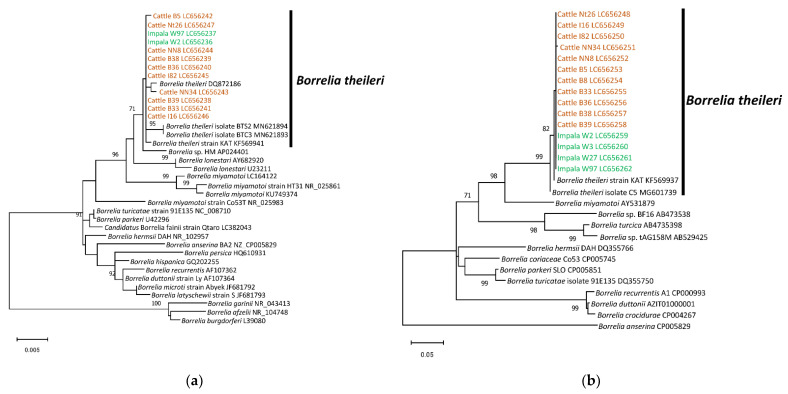
Phylogenetic trees of *Borrelia* spp. based on the sequences of 16S rDNA and *hpt*: (**a**) 16S rDNA; (**b**) *hpt*. The accession numbers for nucleotide sequences are shown after the species names. The analyses were performed using the maximum likelihood method. Bootstrap values > 70% based on 1000 replications are presented on the interior branch nodes.

Similarly, the detected *Borrelia* spp. from impalas and cattle were positioned within the clade of *Borrelia theileri* in the phylogenetic trees based on the almost full-length 16S rDNA and the partial sequence of *hpt* (Figure 2). 

### 3.4. Statistical Analysis

The mean PCV values of *Borrelia* infected and non-infected cattle were 32.1 (standard deviation (SD): 6.16, 95% confidence interval (CI): 29.22–34.98) and 31.9 (SD: 5.81, 95% CI: 31.40–32.46), respectively. There was no significant difference in PCV between infected and non-infected cattle (*p* = 0.74).

## 4. Discussion

In this study, we investigated the presence of *Borrelia* spirochetes in four different wild animal species and domestic cattle in the Kafue ecosystem in Zambia using molecular methods and successfully identified *Borrelia theileri* in impalas and cattle. To the best of our knowledge, this study provides the first evidence of *Borrelia* spirochetes in impalas in Africa and the first report of *Borrelia theileri* in cattle in Zambia.

*Borrelia theileri* is the causative agent of bovine borreliosis, first reported in cattle in South Africa in 1903 [21]. Zambia is a landlocked country located in south central Africa and shares its borders with Zimbabwe, Namibia, Botswana, Mozambique, Malawi, Tanzania, Democratic Republic of Congo, and Angola. Among these surrounding countries, only Botswana has reported cases of bovine borreliosis [41]. However, the previous report did not have any molecular information on the detected *Borrelia theileri*. The present study provides molecular evidence for a new geographical record of *Borrelia theileri* in Zambia. Furthermore, considering that cross-border trade is common in cattle and that wildlife migration occurs between Zambia and its neighbors, *Borrelia theileri* might have spread to the surrounding countries through infected animals.

A previous study in North Cameroon, using PCR, found that 17.9% (225/1260) of cattle were infected with *Borrelia* spp. [42]. However, out of 225 positive cattle, only 42 were confirmed to be infected with *Borrelia theileri* by sequence analysis. Thus, the infection rate of *Borrelia theileri* was 3.3% (42/1260) in the cattle population in North Cameroon. The present study also showed a similar infection rate (4.1%) in cattle to a previous study in North Cameroon. In addition, the present study revealed that 5.1% of the impalas in the Kafue ecosystem were infected with *Borrelia theileri*. However, there have been no studies on the infection rate of *Borrelia theileri* in large and middle-sized wildlife. Nevertheless, previous studies in Japan showed that 10.6% (25/235) and 25.9% (165/638) of wild sika deer were infected with a *Borrelia* sp., similar to *Borrelia lonestari*, which is a closely related species to *Borrelia theileri* [27,43].

Zambia is an endemic region of the East Coast Fever (ECF), a fatal disease in cattle caused by the blood parasite *Theileria parva* [44] and Redwater or bovine babesiosis caused by *Babesia bigemina* and *Babesia bovis* [45]. Another severe cattle disease, African animal trypanosomiasis, caused by *Trypanosoma* spp., is also endemic to Zambia [42]. Animals infected with these diseases have common clinical symptoms, such as fever, anemia, jaundice, hemoglobinuria, and swollen lymph nodes [44,45,46], and these diseases often result in death. Therefore, these diseases are of great veterinary and economic importance, as they have a considerable effect on livestock production [44]. In contrast, bovine borreliosis shows mild but similar symptoms, such as fever, lethargy, and anemia [42]. In this study, *Borrelia* infection was not related to anemia, statistically. Nevertheless, these symptoms can affect cattle performance and reduce their production. Furthermore, the livelihoods of an estimated 700 million rural African poor are supported or maintained by livestock ownership [47]. In addition to its intrinsic value, livestock provide a flow of food and income over time, helping to increase farm productivity; for most rural communities, livestock production is their only available livelihood [47]. Therefore, recognizing the existence of *Borrelia theileri* in Zambia was helpful in the clinical differentiation between bovine borreliosis and other bovine diseases for better treatment and control measures, which would ensure that farmers bring healthier and more valuable products to the market.

Human activities and associated land use changes have caused an increase in proximity between domestic and wild animals in many places, resulting in the mutual exchange of pathogens [20,48]. As is often the case near national parks in Zambia, domestic and wild animals often share a grazing place. In this study, we detected *Borrelia theileri,* which had sequences identical to those of cattle and impalas. Thus, the same strain of *Borrelia theileri* might be circulating between cattle and impalas in the Kafue ecosystem. Furthermore, Espinaze et al. (2018) revealed that large and middle-sized animals were highly connected by the tick species they shared, facilitating cross-infection with ticks and the transmission of tick-borne pathogens, including *Borrelia theileri* [49]. In particular, domestic animals can play an important role in accelerating the spread of ticks and tick-borne pathogens in Southern African animal communities [49], which means that these ticks and tick-borne pathogens could be introduced into the Kafue ecosystem. Therefore, although the pathogenicity of *Borrelia theileri* in wild animals is not known, limiting contact between cattle and wild animals might be a good conservation measure for reducing tick-borne diseases in the Kafue ecosystem. Furthermore, investigations of vector tick species for *Borrelia theileri* in the Kafue ecosystem are required for understanding their natural life cycle in the ecosystem.

In this study, we revealed the presence of *Borrelia theileri*, a causative agent of bovine borreliosis in wild impalas and domestic cattle in the Kafue ecosystem in Zambia. Our findings indicate the circulation of spirochetes between impalas and cattle. Furthermore, bovine borreliosis has veterinary and economic implications in livestock, even though the clinical presentation is not as severe as in other bovine diseases. As the study was conducted with samples collected only from the Kafue ecosystem, further investigations are required to clarify comprehensive *Borrelia theileri* status in the country by expanding the sampling area and including additional wild animal and livestock species, especially ungulates.

## Figures and Tables

**Table 1:** Primers used in the conventional PCRs.

Target Gene	Primer Name	Sequence (5′–3′)	Annealing Temperature	Expected Size	Reference
Flagellin (P41)	BflaPAD ^1^	GATCARGCWCAAYATAACCAWATGCA	50 °C	453 bp	[37]
	BflaPDU ^1^	AGATTCAAGTCTGTTTTGGAAAGC			
	BflaPBU ^2^	GCTGAAGAGCTTGGAATGCAACC	50 °C	347 bp	[37]
	BflaPCR ^2^	TGATCAGTTATCATTCTAATAGCA			
hypoxanthine-guanine phosphoribosyltransferase	hptdegF	GCAGAYATTACAAGAGARATGG	55 °C	433 bp	[38]
	hptdegR	CYTCRTCACCCCATTGAGTTCC			
16S ribosomal DNA	BF1	GCTGGCAGTGCGTCTTAAGC	55 °C	1371 bp	[39]
	BR1	GCTTCGGGTATCCTCAACTC			

^1^ Primer for first PCR. ^2^ Primer for nested PCR.

**Table 2:** Results of each PCR assay.

Species	Real-Time PCR	*flaB*	16S rDNA	*hpt*
Impala	5/97 (5.1%)	4/5	2/5	4/5
Hartebeest	0/37 (0%)	NA	NA	NA
Lion	0/4 (0%)	NA	NA	NA
Wild dog	0/2 (0%)	NA	NA	NA
Cattle	20/488 (4.1%)	16/20	10/20	11/20

NA: Not applicable.

## Data Availability

All relevant data are provided in the manuscript.

## Supplementary Materials

The following are available online at https://www.mdpi.com/article/10.3390/microorganisms9112405/s1, Table S1: Accession numbers of the DNA sequences obtained in this study.
