# Supplementary material for: Evidence of Borrelia theileri in Wild and Domestic Animals in the Kafue Ecosystem of Zambia
Source: Microorganisms. 2021 Nov 22;9(11):2405. doi: 10.3390/microorganisms9112405 (PMC8624021; doi:10.3390/microorganisms9112405)
Supplement: Supplementary file 1 [file microorganisms-09-02405-s001.zip › microorganisms-1474635-supplementary.pdf]

Supplemental Table S1. Accession numbers of the obtained DNA sequences in this study

Species	Sample ID	Ct value	*flaB*	16S rDNA	*hpt*
Impala	W2	27.87	LC656216	LC656236	LC656259
Impala	W3	36	LC656217	n.a.	LC656260
Impala	W27	33.1	LC656218	n.a.	LC656261
Impala	W97	31	LC656219	LC656237	LC656262
Impala	W106	34.16	n.a.	n.a.	n.a.
Cattle	B5	33.12	LC656227	LC656242	LC656253
Cattle	B8	33.26	LC656228	n.a.	LC656254
Cattle	B13	35.03	LC656229	n.a.	n.a.
Cattle	B30	35.65	n.a.	n.a.	n.a.
Cattle	B33	30.7	LC656230	LC656241	LC656255
Cattle	B36	32.79	LC656231	LC656240	LC656256
Cattle	B38	34.32	LC656232	LC656239	LC656257
Cattle	B39	29.11	LC656233	LC656238	LC656258
Cattle	B44	32.22	LC656234	n.a.	n.a.
Cattle	B106	34.66	LC656235	n.a.	n.a.
Cattle	I16	27.6	LC656223	LC656246	LC656249
Cattle	I29	34.94	n.a.	n.a.	n.a.
Cattle	I64	35.9	n.a.	n.a.	n.a.
Cattle	I82	35.26	LC656224	LC656245	LC656250
Cattle	K23	30.31	LC656221	n.a.	n.a.
Cattle	K83	33.98	LC656222	n.a.	n.a.
Cattle	NN8	33.48	LC656225	LC656244	LC656252
Cattle	NN31	35.22	n.a.	n.a.	n.a.
Cattle	NN34	28.52	LC656226	LC656243	LC656251
Cattle	Nt26	29.63	LC656220	LC656247	LC656248

n.a.: not applicable
